# Auxin canalization and vascular tissue formation by TIR1/AFB‐mediated auxin signaling in Arabidopsis

**DOI:** 10.1111/nph.16446

**Published:** 2020-02-22

**Authors:** Ewa Mazur, Ivan Kulik, Jakub Hajný, Jiří Friml

**Affiliations:** ^1^ University of Silesia in Katowice Faculty of Natural Sciences Institute of Biology, Biotechnology and Environmental Protection Katowice Poland; ^2^ Mendel Centre for Plant Genomics and Proteomics Central European Institute of Technology (CEITEC) Masaryk University CZ‐62‐500 Brno Czech Republic; ^3^ Institute of Science and Technology (IST) 3400 Klosterneuburg Austria; ^4^ Laboratory of Growth Regulators and Department of Chemical Biology and Genetics Centre of Region Haná for Biotechnological and Agricultural Research Faculty of Science Palacký University and Institute of Experimental Botany ASCR Šlechtitelů 27 783 71 Olomouc Czech Republic

**Keywords:** *Arabidopsis thaliana*, auxin, auxin canalization, cell polarity, PIN1, TIR1/AFB

## Abstract

- Plant survival depends on vascular tissues, which originate in a self‐organizing manner as strands of cells co‐directionally transporting the plant hormone auxin. The latter phenomenon (also known as auxin canalization) is classically hypothesized to be regulated by auxin itself via the effect of this hormone on the polarity of its own intercellular transport. Correlative observations supported this concept, but molecular insights remain limited.
- In the current study, we established an experimental system based on the model *Arabidopsis thaliana*, which exhibits auxin transport channels and formation of vasculature strands in response to local auxin application.
- Our methodology permits the genetic analysis of auxin canalization under controllable experimental conditions. By utilizing this opportunity, we confirmed the dependence of auxin canalization on a PIN‐dependent auxin transport and nuclear, TIR1/AFB‐mediated auxin signaling. We also show that leaf venation and auxin‐mediated PIN repolarization in the root require TIR1/AFB signaling.
- Further studies based on this experimental system are likely to yield better understanding of the mechanisms underlying auxin transport polarization in other developmental contexts.

## Introduction

Plants possess superb abilities to adapt their development to the changing environment. One of them is their capacity to form organized vasculature, which occurs under normal (e.g. leaf venation or when nascent organs connect to the pre‐existing vascular network) and traumatic (e.g. re‐connection of broken vascular strands after wounding) conditions. The latter example occurs frequently within the ontogeny of higher plants (due to grazing or other types of mechanical stress) and is therefore paramount to their survival. This developmentally fascinating process of vasculature formation involves not only (de)differentiation of multiple cell types, but also coordinated cell polarization ultimately leading to the directional transport of compounds through cellular strands (channels).

It has been proposed that vascular strand formation is regulated by auxin via a putative feedback interaction between its cellular perception and intercellular polar transport (Sachs, 1975, 1981; Uggla *et al.*, 1996, 1998; Tuominen *et al.*, 2000; Sauer *et al.*, 2006; Robert *et al.*, 2013). It is known that vasculature formation is indeed spatially associated with the activation of TIR1/AFB signaling (Lavy *et al.*, 2016) and accumulation of polarly distributed PIN‐FORMED (PIN) auxin efflux proteins (Adamowski & Friml, 2015) in the co‐directionally polarized strands of vascular progenitors (auxin canalization; Sauer *et al*, 2006; Zhang *et al.*, 2010; Balla *et al.*, 2011; Mazur *et al.*, 2016; Prat *et al.*, 2018). Observations of a similar correlation between auxin signaling and auxin transport polarization have also been made during embryonic apical–basal axis establishment (Robert *et al.*, 2013), shoot and root organogenesis (Benkova *et al.*, 2003; Heisler *et al.*, 2005; Bhatia *et al.*, 2016) as well as unexpected process such as the termination of shoot gravitropic response (Rakusova *et al.*, 2016).

The classical, ‘gold standard’ cell biological studies on auxin canalization were based on local auxin application onto the tissues of different plant species (Raven, 1975; Sachs, 1975, 1981), including pea (*Pisum sativum*) stems (Sauer *et al.*, 2006; Balla *et al.*, 2011). In this setup, auxin‐transporting channels (and subsequently vascular strands) developed from the application site and connected it to the pre‐existing vasculature of a plant. While these observations indicated that auxin canalization occurs via self‐organization rather than pre‐patterning, further implementation of the classical methodology has been hampered by the difficulty of transgenesis in the corresponding plant species.

Previous reports (Berleth *et al.*, 2000; Dettmer *et al.*, 2009; Bennett *et al.*, 2014) suggested that auxin canalization also underlies physiological processes such as vasculature regeneration after wounding (Sauer *et al.*, 2006; Mazur *et al.*, 2016), leaf venation (Scarpella *et al.*, 2006; Cano‐Delgado *et al.*, 2010; Sawchuk & Scarpella, 2013) and auxin‐mediated PIN lateralization in the root (Prat *et al.*, 2018). In particular, it has been shown that leaf vein specification is the result of directional auxin transport mediated by polarized PIN expression demarcating the position of future vascular patterning. From primary broader PIN1 expression domains, the narrow PIN1‐marked routes of auxin transport emerged as polarized groups of cells differentiating into vascular connections in leaves (Scarpella *et al.*, 2006; Wenzel *et al.*, 2007). Thus, studies utilizing the classical experimental model based on local auxin application are likely to yield knowledge not only on auxin canalization in the context of its exogenous application but also in other, more physiological roles.

Which components of auxin perception are involved in its feedback on auxin transport has not been rigorously addressed, but the well‐characterized signaling pathway involving TRANSPORT INHIBITOR RESPONSE1 (TIR1)/AUXIN SIGNALING F‐BOX (AFB) proteins as auxin receptors and the downstream Aux/IAA and auxin response factor (ARF) transcriptional regulators are likely to be implicated (Dharmasiri & Estelle, 2004; Hayashi *et al*, 2012). Although the molecular mechanisms are not entirely clear, it was shown that downstream processes in leaf vascular patterning are controlled by the auxin response transcription factor MONOPTEROS (MP) through an auxin response element in the *AtHB8* gene promoter. *AtHB8* seems to be required to constrict cell fate acquisition to gradually narrower areas, leading to the establishment of procambial cell identity during vein development (Donner *et al.*, 2009). Nonetheless, a demonstration that TIR1/AFB nuclear auxin signaling is required for the auxin feedback on auxin transport polarization during canalization, thus regulating processes such as leaf venation and wounding‐induced vasculature regeneration, has not been provided.

Here, we established an experimental system, in which auxin canalization and vasculature formation can be induced by local auxin application. This makes the setup more direct and controllable compared to our previous approach, which involved vasculature regeneration around the wound (Mazur *et al.*, 2016). We use this system in conjunction with genetic, pharmacological and cell biological methods to demonstrate the requirement of TIR1/AFB signaling for auxin canalization and also show its importance for the regeneration of vascular strands and leaf venation.

## Materials and Methods

### Plant material and plant growth conditions

Wild‐type Col‐0 (NASC, The Nottingham Arabidopsis Stock Centre; http://www.arabidopsis.info/BasicForm) and reporter lines *DR5rev::GFP* (Friml *et al.*, 2003) and *pPIN1::PIN1:GFP* (Benkova *et al.*, 2003) produced in the Col‐0 background were used as controls. *pin1‐1*, *tir1‐1*, *tir1‐1 afb2 afb3*, *arf7‐394 arf19‐1* and *HS::axr3‐1* have been previously described (Knox *et al.*, 2003; Sauer *et al.*, 2006; Lavy & Estelle, 2016; Fendrych *et al*, 2016). *tir1‐1 afb1 afb3* was produced by us for this study. All mutants and transgenic lines used in this study are in the *Arabidopsis thaliana* ecotype Columbia (Col‐0) background. Plants were germinated in pots with soil and vermiculite mixture (1 : 1, v : v). Seedlings with two pairs of true leaves were individually planted and grown in pots with soaked peaty rings in a growth chamber under long‐day light conditions at 20°C. Plants with inflorescence stems 10 cm tall were chosen for the experiments.

### Local auxin application and vasculature regeneration experiments in Arabidopsis stems

Young plants with inflorescence stems having primary tissue architecture (vascular bundles separated by interfascicular parenchyma sectors) were chosen for the following two‐step experiments, according to the protocol of Mazur *et al. *(2016). First, the flowering parts of the stems were removed by using a sharp razor blade. The resulting stems (7 cm tall after dissection) were attached to a polypropylene tube to stiffen them and placed under a lead ball (2.5 g). The weight was applied for 6 d to produce a closed ring of cambium on the stem circumference (Mazur *et al.*, 2014). Next, the samples were incised transversally above the leaf rosette, and a droplet of lanoline paste with auxin (IAA; Sigma‐Aldrich, cat. no. 15148‐2G) or auxin plus inhibitors (NPA (*N*‐1‐naphtylphthalamic acid), Sigma; PEO‐IAA (α‐(phenyl ethyl‐2‐one)‐indole‐3‐acetic‐acid; auxinole, Sigma)) was locally applied below the cut. The incision was made in the transverse plane to disturb the longitudinal continuum of cambium and polar, basipetal transport of endogenous auxin. We were thus certain that the analyzed changes are the results of the externally applied auxin only. The applied compounds were replaced during the experiments every 2 d with a fresh droplet. For local application, 10 µM water solutions of all compounds mixed with a droplet of lanolin paste were used. Stock solutions of auxin and inhibitors (NPA, auxinole, PEO‐IAA) were dissolved in dimethyl sulfoxide (cat. no. D5879‐500ML; Sigma). Experiments were conducted twice for each line, with at least 10 plants analyzed in each run. Finally, the samples were collected, manually sectioned and mounted in a 50% glycerol aqueous solution onto imaging glass.

### Leaf and cotyledon clearing

To reveal their vasculature, leaves/cotyledons of 8‐d‐old plants were treated with the following: 70% ethanol (overnight at 4°C); 4% HCl + 20% methanol (12 min at 65°C); 7% NaOH + 60% ethanol (15 min at room temperature); HCl (10 min at room temperature); seedlings were rehydrated by successive incubations in 60/40/20/10% in ethanol for 10 min; and 5% ethanol + 25% glycerol (a few days at 4°C until the air bubbles within the tissue had disappeared).

### Verification of transgenic line identity

The mutations in the genomes of the mutant plant lines used in this work were verified by PCR. Namely, genomic DNA was extracted from mechanically ground leaves of 3‐wk‐old plants and used as a template in PCR with wild type‐ and mutation‐specific primers. The presence of the *tir1‐1* point mutation was tested via *tir1* amplicon digestion with *Mbo*I endonuclease. Since regular PCR results were inconclusive for *afb1* and *afb3* insertional mutations in the *tir1afb1afb3* mutant, reverse transcription PCR was used instead, with cDNA from total leaf RNA preparation used as a template.

### Imaging and image analysis

Samples of wounded stems were analyzed via a stereomicroscope (Nikon MSZ1500) equipped with a charge‐coupled device (CCD) camera DS‐Fi1. The green fluorescent protein (GFP) reporter lines were analyzed using Zeiss Observer.Z1 and Olympus Fluoview FV1000 confocal laser‐scanning microscopes. GFP fluorescence was excited by an argon‐ion laser light of 488 nm, detected at 510 nm. Acquired images were processed with ZEN 2012 Light Edition and fluoview software. Transmitted light observations were made via an Olympus BX43 microscope equipped with Olympus SC30 camera. Figures were created with coreldraw X6.

### Quantification and statistical analysis

All calculations and graphs were made with Microsoft Office excel software. Unpaired Student's *t*‐tests (*P* < 0.05) and one‐way ANOVA were used to compare sets of data (*P* < 0.0001). Error bars in the graphs indicate standard errors.

## Results

### Vasculature regeneration after wounding requires TIR1/AFB signaling

Previously (Mazur *et al.*, 2016), we showed that stem vasculature regeneration after wounding was associated with the activation of nuclear auxin signaling and induction of PIN1 auxin transport channels. In the present work, we wanted to test if this regeneration was dependent on the latter two factors.

**Figure 1:**
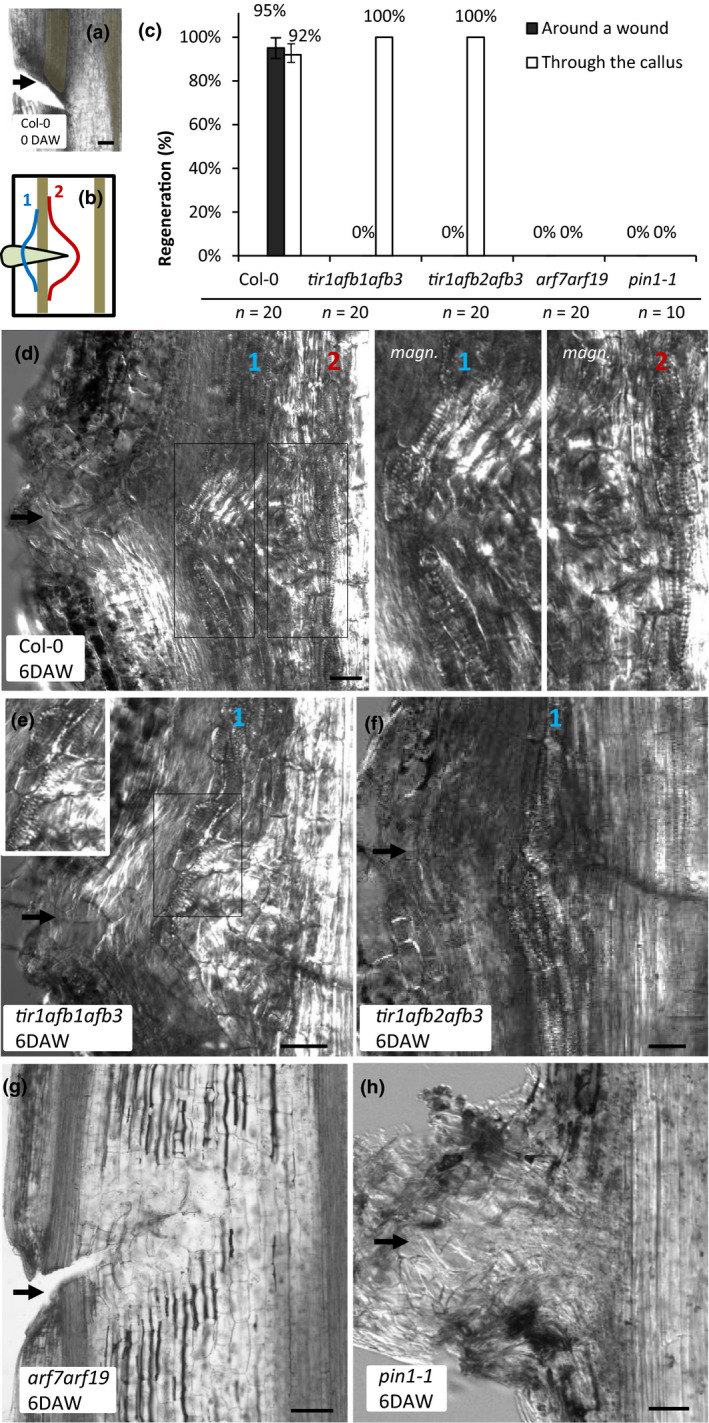
Vasculature regeneration after wounding in *tir1/afb*, *arf7arf19* and *pin1‐1* mutants of Arabidopsis. (a) Longitudinal section through a Col‐0 inflorescence stem 8 h after wounding before callus formation. Black arrow indicates the approximate location of the wound. (b) Schematic depiction of vasculature regeneration after wounding in Arabidopsis inflorescence stem. Two brown vascular strands are shown similar to (a). The left one is broken due to wounding. The wound is filled with callus (light brown). (c) Frequencies of vasculature regeneration after wounding in stems of different genotypes. Error bars indicate SE. (d–h) Examples of wounded *Col‐0*, *tir1afb1afb3*, *tir1afb2afb3*, *arf7arf19* and *pin1‐1* inflorescence stems 6 d after wounding (DAW). Black arrows show the approximate locations of wounding sites. White rectangles specify which parts of the images are magnified to make the secondary cell wall features of regenerated vascular cells more apparent. Blue (‘1’) and red (‘2’) numbers mark the regenerated vascular strands passing through callus and around the wound, respectively. Bars, 50 µm (a, d–h). See also Supporting Information Fig. S1.

To this end, we wounded inflorescence stems of Col‐0 as well as triple *tir1afb1afb3* and *tir1afb2afb3* mutants and assessed the extent of vasculature regeneration in them 6 d after wounding (DAW; Fig. 1a,b). Double ARF (*arf7arf19*) mutants were analyzed as well, because these particular ARFs have been shown previously to be required for auxin signaling and auxin‐mediated re‐arrangements of polar PIN1 distribution in roots (Okushima *et al.*, 2005; Sauer *et al.*, 2006). While two modes of vascular strand formation, namely passing around the wound and through the callus forming within the wound (both composed of elongated cells with stripes of secondary cell wall features) were present in the majority of sectioned Col‐0 stems, only the vasculature passing through callus was visible in all *tir*/*afb* triple mutant samples (Fig. 1c–f). All *arf7arf19* mutant samples lacked callus formation after injury and did not regenerate vasculature around the wound (Fig. 1g).

In addition to these constitutive mutants, we analyzed *HS::axr3‐1* plants, in which the expression of a dominant negative form of the IAA17/AXR3 transcriptional repressor can be induced by thermal stress (Knox *et al.*, 2003; Hayashi, 2012). After the stems were wounded, *HS::axr3‐1* induction was conducted by incubating the plants at 37°C for 1 h every day, which strongly inhibited vasculature regeneration and callus formation. In particular, no vasculature formed around the wound in 70% (14/20) of samples 6 DAW. In the remaining 30% of stems (6/20), groups of cells with denser cell walls were present above the wound 6 DAW (Supporting Information Fig. S1). They were never elongated or arranged into well‐defined strands and lacked the signs of secondary cell wall patterning.

To study the importance of PIN1, the same experiments were conducted on *pin1‐1* knockout plants. The results were similar, with no vasculature passing through callus or around the wound visible 6 DAW in all samples (Fig. 1h).

Thus, our analysis of vasculature regeneration after wounding in constitutive and inducible mutants revealed that while PIN1‐mediated auxin transport and TIR1/AFB auxin perception are required for vasculature regeneration around the wound, vasculature can still regenerate through callus when either one of these two processes is suppressed.

### Local auxin application induces TIR1/AFB‐ and PIN1‐dependent vascular strand development in Arabidopsis

**Figure 2:**
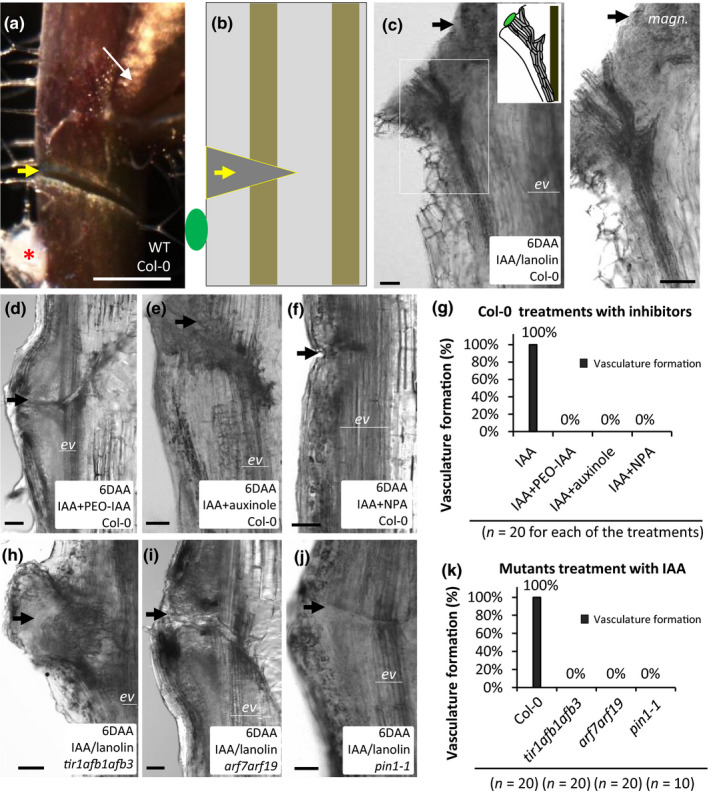
Vascular strands formation in response to local auxin application onto Arabidopsis stems. (a) Fragment of a wounded Col‐0 inflorescence stem. Auxin (with or without various inhibitors) was locally applied in a droplet of lanolin paste below the wound (red asterisk). Axillary buds above the wound were not removed in the experiments (narrow white arrow). (b) Schematic representation of (a) on a longitudinal section. Compounds are applied in a droplet of lanolin paste below the wound (green oval). The droplet is removed from the sample before sectioning for technical reasons and therefore cannot be located on the actual images. The vascular strand is broken by a transverse cut. (c–f) Examples of longitudinal sections of Col‐0 stems 6 d after local application (DAA) of IAA alone or its co‐application with PEO‐IAA, auxinole or NPA. (h–j) Examples of longitudinal sections of *tir1afb1afb3*, *arf7arf19* and *pin1‐1* stems 6 d after local application of IAA. (g, k) Frequencies of vascular strands formation under the studied experimental conditions. Thick arrows (yellow or black) indicate the approximate location of wounds. The bottom fragments of broken vascular strands (pre‐existing vasculature) are labeled ‘ev’. Bars: (a) 1mm; (c–f, h–j) 50 µm.

Having observed a failure of *tir1/afb* mutants to regenerate vasculature after wounding, we wanted to test if this was due to the direct involvement of TIR1/AFB auxin perception in auxin canalization rather than in other regeneration‐associated processes. For this, we complemented our wounding protocol with local auxin application. This is a classical experimental setup, which allows us to induce formation of auxin channels specifically by auxin treatment. In particular, although wounding is required in these experiments to stop the normal flow of auxin, its canalization is not induced unless the injury is accompanied by local auxin application below the cut site (Fig. 2a,b).

Application of natural auxin (IAA) dissolved in lanolin wax (100 nM) onto the surface of wounded Col‐0 inflorescence stems led to the formation of thick vasculature (appearing as black strands extending downwards from the periphery into the deeper regions of the tissue) 6 d after auxin application (DAA) in almost 80% (32/40) of samples (Fig. 2c).

To test if this phenomenon depended on TIR1/AFB signaling and PIN1‐mediated auxin transport, we treated wounded Col‐0 stems with local co‐application of auxin and inhibitors of either TIR1/AFB signaling (auxinole, PEO‐IAA) or auxin transport (NPA) in the same drop of wax (Hayashi *et al.*, 2012). No vascular strands developed around the auxin application site 6 DAA under such experimental conditions (Fig. 2d–g). Local application of auxin onto the stems of *tir1afb1afb3*, *arf7arf19* and *pin1‐1* mutants yielded similar results (Fig. 2h–k).

Thus, via a combination of genetic and pharmacological approaches, we show that TIR1/AFB signaling and PIN1‐dependent auxin transport are required for vascular strands development in response to local auxin application in Arabidopsis inflorescence stems.

### Local auxin application induces TIR1/AFB‐ and PIN1‐dependent auxin canalization in Arabidopsis

To validate that vascular strand formation in response to local auxin application represents auxin canalization, we visualized auxin response and polar auxin transport in the established experimental system via the genetic markers* DR5rev::GFP* (Friml *et al.*, 2003) and p*PIN1::PIN1‐GFP* (Benkova *et al.*, 2003), respectively.

**Figure 3:**
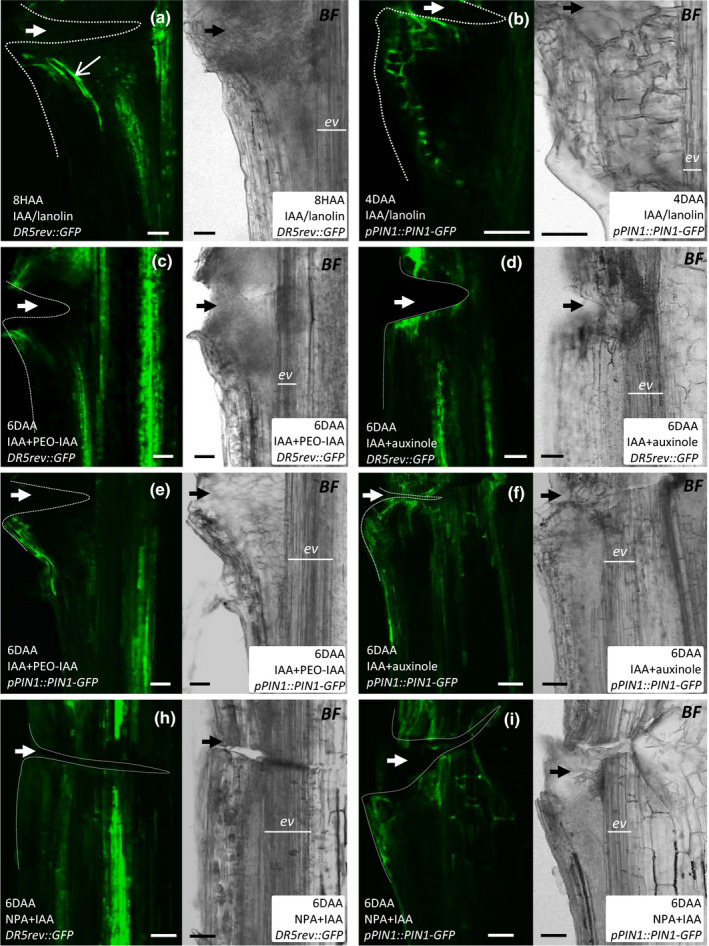
Requirement of TIR1/AFB auxin perception and PIN‐mediated auxin transport in auxin canalization induced by local auxin application onto Arabidopsis stems. (a–i) Examples of longitudinal sections through *DR5rev::GFP* and *pPIN1::PIN1‐GFP* stems obtained after local application of IAA alone or along with PEO‐IAA, auxinole or NPA. Each section was imaged in green and bright field channels. Dotted lines indicate the wounded stem regions. Thick arrows (white or black) indicate the approximate location of wounds. Bars, 50 µm. See also Supporting Information Fig. S2.

Local auxin application resulted in DR5 activation at the application site 8 h after auxin application (HAA; Fig. 3a). GFP‐positive cells were arranged in a strand, similar to the vasculature in Fig. 2(c). At 4 DAA, a wide field of bright GFP fluorescence (Fig. S2a) and a narrow channel of cells expressing PIN1‐GFP (Fig. 3b) were observed between the organ periphery and the pre‐existing stem vasculature. Much weaker induction of DR5rev::GFP and PIN1‐GFP expression was visible 4 and 6 DAA near the application site, when the stems were locally co‐treated with IAA and TIR1/AFB inhibitors or NPA (Fig. 3c–i; Fig. S2b,c). In particular, green cells did not form defined strands under these experimental conditions and instead were found at the periphery of the organ.

Thus, these data show that local auxin application onto Arabidopsis inflorescence stems induces formation of PIN1‐positive, high‐auxin response channels from the exogenous source towards the pre‐existing stem vasculature, which is blocked by pharmacological inhibition of either auxin perception or its directional transport.

### TIR1/AFB signaling is required for proper leaf venation and auxin‐induced PIN1 lateralization in the root

To complement our wounding and local auxin application observations with less invasive experiments, we analyzed other, spontaneously occurring auxin canalization‐related physiological processes.

**Figure 4:**
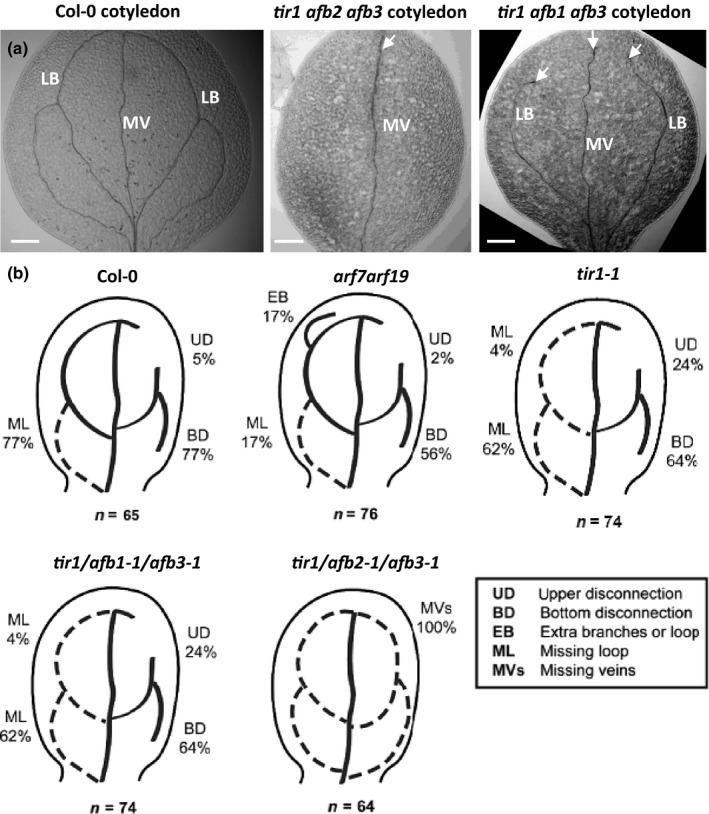
Cotyledon venation in TIR1/AFB mutants of Arabidopsis. (a) Vasculature defects in Col‐0, *tir1afb2afb3* and *tir1afb1afb3* cotyledons. Arrowheads highlight vasculature defects of the lateral branches (LB) and the middle vein (MV). (b) Schemes demonstrating the types and frequencies of venation abnormalities observed. Bars, 200 µm (a). See also Supporting Information Figs S3 and S4.

First, we looked at leaf venation, because it requires PIN‐dependent auxin transport and is accompanied by the formation of DR5/PIN1‐positive channels (Scarpella *et al.*, 2006; Sawchuk & Scarpella, 2013), similar to the case of auxin canalization from the exogenous source. We observed that two triple mutants defective in TIR1/AFB‐mediated auxin perception (*tir1afb1afb3* and *tir1afb2afb3*) exhibited strong leaf venation defects in cotyledons (Fig. 4a) and primary leaves (Fig. S4). The strong abnormalities included apical disconnections between the central and lateral veins in cotyledons and the lack of one or both lateral veins in leaves (Fig. 4b). Thus, the analysis of leaf/cotyledon vasculature in *tir1/afb* mutants evidently shows that although vasculature can form, its intricate, organized pattern during leaf venation strongly depends on TIR1/AFB auxin perception.

To obtain some glimpses into which cellular processes during canalization are targeted by TIR1/AFB signaling, we analyzed PIN1 re‐localization in the root tip cells occurring in response to a relatively short (4 h) auxin treatment (Sauer *et al.*, 2006; Prat *et al.*, 2018). This phenomenon has an unclear physiological significance but provides a simple assessment of auxin's effect on PIN polarity – one of the key prerequisites of canalization. Notably, it also asseses this effect without any obvious accompanying morphological changes or cell fate re‐specification processes occurring during vasculature formation. Normally in this case, the intracellular localization of PIN1 shifts from a predominantly basal position to the inner lateral side of endodermal and pericycle cells. However, we found the roots of *tir1afb2afb3* plants to be much less responsive to auxin in terms of this PIN1 lateralization (Fig. S3a,b), which is consistent with similar, previously published observations in *axr3* and *arf7arf19* mutants (Sauer *et al.*, 2006).

In summary, these observations demonstrate that TIR1/AFB signaling is important for auxin canalization not only under more invasive experimental conditions, such as wounding and local auxin application, but also in an undisturbed, physiological process involving vasculature formation such as leaf venation. The TIR1/AFB signaling may act on auxin‐mediated PIN1 repolarization, as suggested by defects in this process in the roots of mutants defective in this auxin signaling pathway.

## Discussion

The vascular tissue network crucially aids plants to thrive in almost all land habitats. The mechanism of its formation is intriguing not only due to the importance of vasculature for plant life altogether, but also due to its reiterative nature and developmental flexibility (it can be induced in many contexts, such as during generation of nascent organs or regeneration after wounding). These properties of vasculature formation are explained by a self‐organizing nature of auxin canalization – an organized establishment of auxin transport channels from localized auxin sources. The most evident manifestation of auxin canalization can be observed in classical experiments involving the induction of a canalized auxin flow away from the site of its local application (Sachs, 1975, 1981; Berleth & Sachs, 2001; Sauer *et al.*, 2006; Balla *et al.*, 2011; Sawchuk & Scarpella, 2013; Bennett *et al.*, 2014; Adamowski & Friml, 2015; Cieslak *et al.*, 2015; Mazur *et al.*, 2016). This process is known to involve intracellular polarization of PIN auxin transporters, which is coordinated between individual vasculature progenitors in a way that ultimately generates auxin‐transporting channels. Details of the molecular and cell biological mechanisms of this coordinated polarization are missing, however. The classical canalization hypothesis proposes the existence of a positive feedback interaction between auxin perception and the regulation of its intercellular transport direction as determined by the cellular PIN polarities (Vieten *et al.*, 2005; Adamowski & Friml, 2015).

In the past, verification of this idea was difficult because of the limitations of transgenesis in the species where local auxin application experiments were possible. In the present study, we demonstrate that the classical methodology can be successfully applied to the classical genetic model *A. thaliana* with the available large collection of mutants and marker lines. The local auxin application experimental setup has several significant advantages over that previously used to study canalization, such as vasculature regeneration after wounding, as it allows us to: exclude the potential confounding factors associated with stem wounding (because the induction of auxin canalization is achieved by auxin application per se); control the dosage of the inductive stimulus (auxin concentration and duration of its supplementation); and test the inductive potential of various auxin analogs and/or complement the induction of auxin canalization with the effects of other bioactive compounds (e.g. inhibitors).

In addition, we show that auxin canalization under the improved experimental setup depends strictly on PIN1‐mediated transport and TIR1/AFB signaling, providing a necessary demonstration of their presumed involvement. The fact that TIR1/AFB signaling is also required for proper progression of auxin canalization‐related processes under more physiological conditions, such as vasculature regeneration after wounding, leaf venation and PIN1 lateralization in the root, suggests that the results derived from the proposed methodology are likely to be not idiosyncratic but of general relevance.

That being said, it would be interesting to test if other cases of vasculature development, such as those occurring during graft transplantation (Melnyk *et al.*, 2015) and organ regeneration from cell culture (Kareem *et al.*, 2015), share this requirement. Furthermore, our experimental system may be used to characterize the role of those auxin signaling components, which have been reported to be important for leaf venation (Donner *et al.*, 2009), under simpler and more controllable auxin canalization conditions.

At the same time, the suggested methodology has certain limitations. In particular, it provides no dynamic, live information, as the samples, due to their thickness and opacity, need to be mechanically sectioned to allow microscopy. For the same reason, it is not trivial to characterize the complete 3D distribution of vasculature and fluorescent reporters, although a more refined method of sectioning compared to that used in this research should alleviate this restriction.

With the present work, we hope to re‐ignite the classical studies of auxin canalization using modern transgenesis and imaging techniques. In particular, hypotheses on the interaction between auxin perception and its polar transport could be tested via local auxin application in particular mutants. For example, various cellular processes such as endocytosis protein recycling or degradation may be genetically and pharmacologically manipulated to probe their role in auxin canalization (Wabnik *et al.*, 2010; Grones *et al.*, 2015). Although not perfect, we think that the suggested methodology would help towards the understanding of how individual plant cells communicate with one another to achieve coordinated tissue polarization and how the auxin‐transporting channels activate the downstream developmental programs of vasculature differentiation.

## Author contributions

EM, IK, JH and JF designed and conducted experiments and analyzed the data. EM and JF wrote the manuscript, with the assistance of IK.

## Supporting information


**Fig. S1 **Defects in vasculature regeneration in wounded *HS::axr3‐1* Arabidopsis mutant.
**Fig. S2** *DR5rev::GFP* and *pPIN::PIN1‐GFP* fluorescence distribution at additional time points after local compound application.
**Fig. S3** PIN1 lateralization in the roots of *Col‐0*, *HS::axr3‐1*, *arf7arf19* and *tir1afb2afb3* genotypes in response to NAA treatment.
**Fig. S4** Abnormal venation in primary leaf of *tir1afb2afb3* Arabidopsis mutant.
Please note: Wiley Blackwell are not responsible for the content or functionality of any Supporting Information supplied by the authors. Any queries (other than missing material) should be directed to the *New Phytologist* Central Office.
