# Supplementary material for: Auxin canalization and vascular tissue formation by TIR1/AFB‐mediated auxin signaling in Arabidopsis
Source: New Phytol. 2020 Feb 22;226(5):1375–83. doi: 10.1111/nph.16446 (PMC7318144; doi:10.1111/nph.16446)
Supplement: Supplementary file 1 — Fig. S1 Defects in vasculature regeneration in wounded HS::axr3‐1 Arabidopsis mutant. Fig. S2 DR5rev::GFP and pPIN::PIN1‐GFP fluorescence distribution at additional time points after local compound application. Fig. S3 PIN1 lateralization in the roots of Col‐0, HS::axr3‐1, arf7arf19 and tir1afb2afb3 genotypes in response to NAA treatment. Fig. S4 Abnormal venation in primary leaf of tir1afb2afb3 Arabidopsis mutant. Please note: Wiley Blackwell are not responsible for the content or functionality of any Supporting Information supplied by the authors. Any queries (other than missing material) should be directed to the New Phytologist Central Office. [file NPH-226-1375-s001.pdf]

New Phytologist **Supporting Information**

Article title: **Auxin Canalization and Vascular Tissue Formation by TIR1/AFB-Mediated Auxin Signaling in Arabidopsis**

Authors: Ewa Mazur, Ivan Kulik, Jakub Hajný, Jiří Friml

Article acceptance date: 10 January 2020

The following Supporting Information is available for this article:

Fig. S1. Defects in vasculature regeneration in wounded *HS::axr3-1* mutant of Arabidopsis.

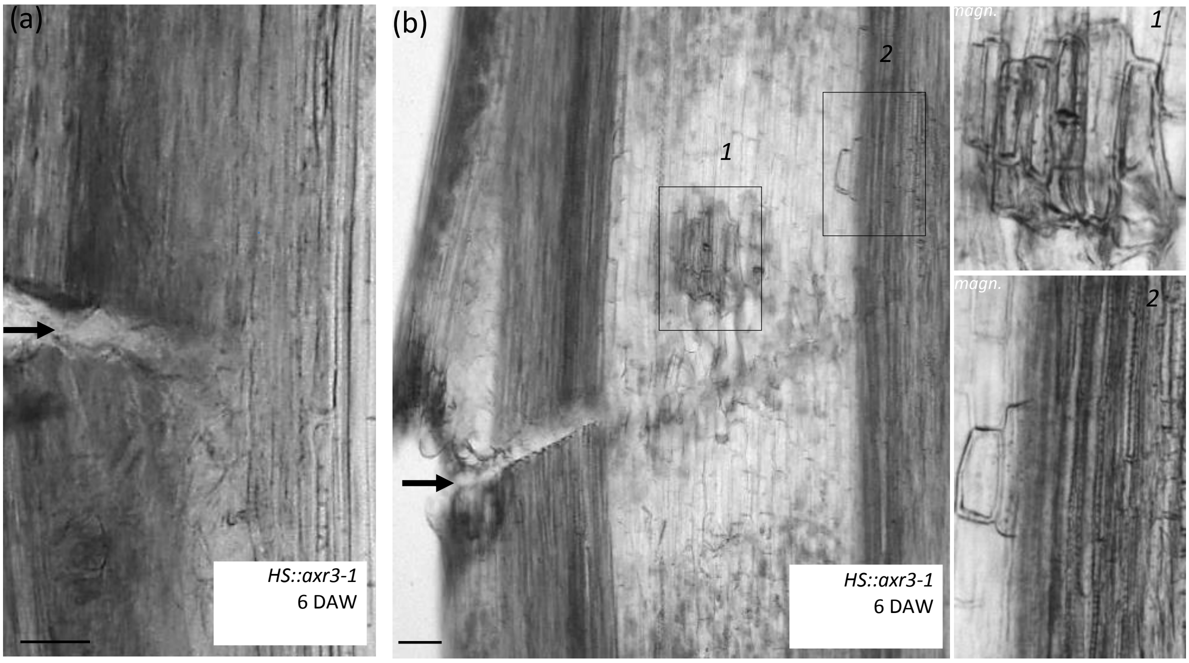

(a) Representative longitudinal section of the samples, in which no vascular cell formation is visible 6 days after wounding.

(b) Representative longitudinal section of the samples, in which limited vascular cell development in visible 6 days after wounding. Two groups of cells that appear to be vascular precursors are highlighted with black squares (and numbers). These regions are magnified.

Black arrows show the approximate location of the wounds. Scale bars represent 50 µm.

Fig. S2. *DR5rev::GFP* and *pPIN::PIN1-GFP* fluorescence distribution at additional time points after local compound application.

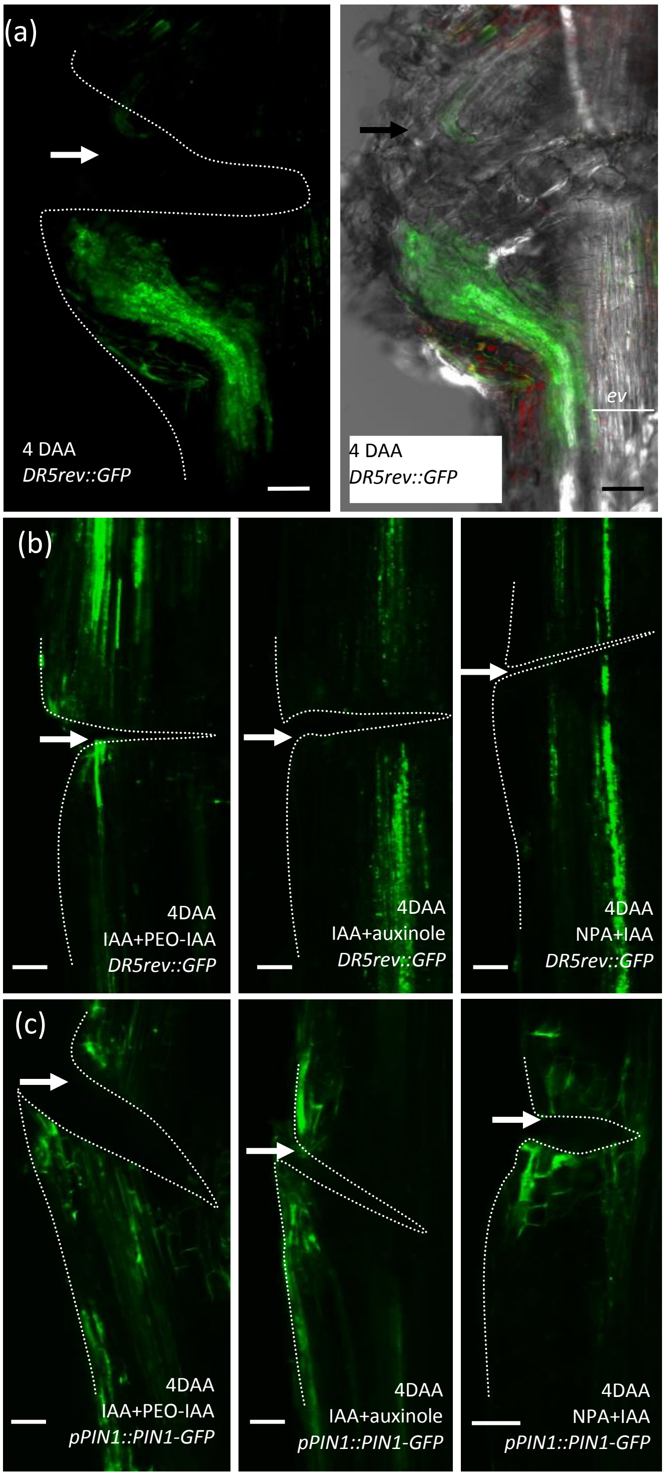

Arrows (white and black) and dotted lines indicate wounded stem regions. Scale bars represent 50 µm.

Fig. S3. PIN1 lateralization in the roots of *Col-0*, *HS::axr3-1*, *arf7arf19* and *tir1afb2afb3* genotypes in response to NAA treatment.

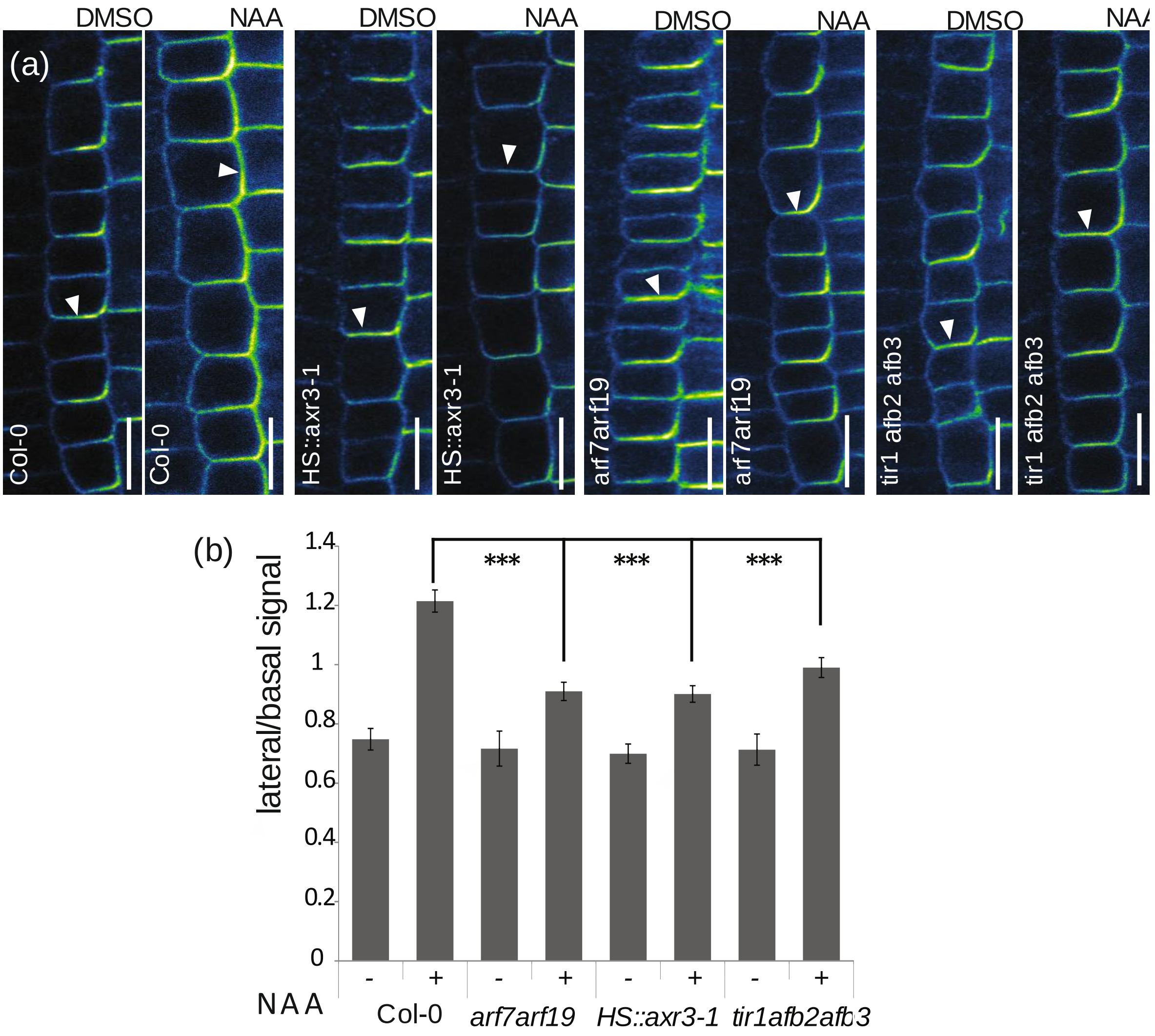

(a) Antibody staining of PIN1 in *HS::axr3-1*, *arf7arf19* and *tir1afb2afb3* mutants in root endodermis cells. Arrowheads highlight PIN1 polarity.

(b) Quantitative evaluation of (a) showing mean ratio of lateral-to-basal signal intensity of PIN1 in endodermis cells. Error bars indicate standard error. A One-Way ANOVA test compared marked sets of data (*** $p<0.0001$; $n>40$ cells corresponding to a minimum of 10 roots per treatment and were imaged under comparable conditions). Experiments were carried out at least 3 times; all quantification of the lateralization was done on the randomized images blindly; photographs from one representative experiment are shown.

Scale bars represent 50 µm.

Fig. S4. Abnormal venation in primary leaf of *tir1afb2afb3* mutant of Arabidopsis.

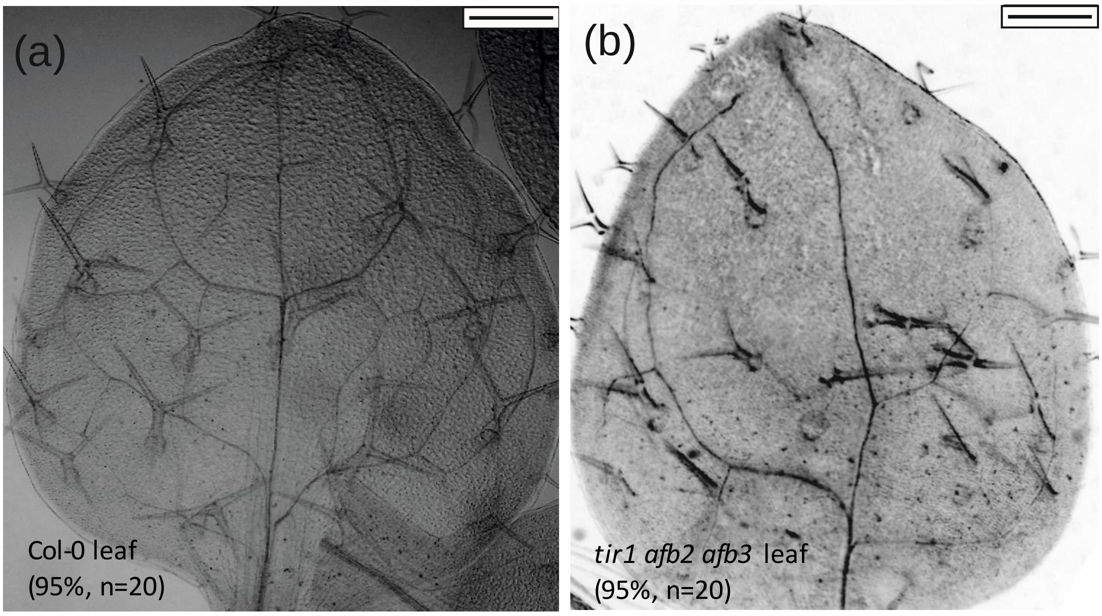

(a) normal venation of Col-0 primary leaf.

(b) strong leaf venation defects in *tir1afb2afb3*. The middle vein development is visible.

Scale bars represent 200 μm.
